# Neighborhood-based PA and its environmental correlates: a GIS- and GPS based cross-sectional study in the Netherlands

**DOI:** 10.1186/s12889-018-5086-5

**Published:** 2018-02-09

**Authors:** Marijke Jansen, Carlijn B. M. Kamphuis, Frank H. Pierik, Dick F. Ettema, Martin J. Dijst

**Affiliations:** 1 Department of Human Geography and Spatial Planning, Faculty of Geosciences, Utrecht University, Heidelberglaan 2, 3584 CS Utrecht, The Netherlands; 2 Department of Sustainable Urban Mobility and Safety, TNO, P.O. Box 80015, 3508 TA Utrecht, The Netherlands

**Keywords:** Neighborhood-based physical activity, GPS, Accelerometer, GIS, Objective neighborhood characteristics

## Abstract

**Background:**

To improve our understanding of the neighborhood environment – physical activity (PA) relationship, it is of importance to assess associations between neighborhood environmental characteristics and neighborhood-based PA.

**Methods:**

Participants’ (*N* = 308; 45–65 years) light PA (LPA) and moderate-vigorous PA (MVPA) within a 400, 800, and 1600 m buffer around adults’ homes was measured using accelerometers and GPS-devices. Land use data in ArcGIS provided neighborhood characteristics for the same buffers. Multilevel linear regression models, adjusted for socio-demographic variables and attitude towards PA, were used to assess associations of objective neighborhood characteristics with neighborhood-based LPA and MVPA.

**Results:**

LPA was positively associated with the proportions of roads (within a 400 m buffer), and negatively associated with the proportions of recreational areas (within an 800 m buffer), and the proportion of green space (within the 800 m and 1600 m buffers). Multiple characteristics of 400 m buffers were positively associated with MVPA, i.e. proportions of green space, blue space, residences, shops and foodservice industry, sports terrain, and public social-cultural facilities. Also, characteristics of larger buffers were positively associated with MVPA, i.e. the proportions of shops and foodservice industry, sports terrain, and blue space (within an 800 m buffer), and the proportion of public social-cultural facilities (within the 800 m and 1600 m buffers).

**Conclusions:**

Objective neighborhood characteristics of smaller as well as larger sized buffers were associated with neighborhood-based LPA and MVPA. Green and blue spaces seem to be of particular importance for PA in the smallest buffer, i.e. in the direct surrounding of adults’ homes.

## Background

Regular physical activity (PA) positively affects health [1, 2]. To achieve health benefits from PA, the World Health Organization (WHO) recommends adults to engage in at least 150 min of moderate PA, or 75 min of vigorous PA per week, or an equivalent combination of both [3]. Worldwide 31.1% of the adult population is insufficiently physically active [4], and increasing population levels of PA is of great importance for population health. However, it is not only moderate-vigorous PA (MVPA) that is of importance for health. Over the past years, researchers have increasingly emphasized the importance for activities of daily living (e.g. household activities, walking, and gardening) or light intensity activities (LPA) as well [5–7]. To adequately inform policy makers, intervention developers and urban planners in designing PA-friendly environments that facilitate both LPA and MVPA, it is important to better understand the relationship between environmental characteristics and PA.

Daily life activities, including PA, take place in many different places (see e.g. [8]). Therefore, throughout the day, individuals are exposed to various environments that have different characteristics. One environment of interest is the residential environment. This environment is one of the daily life environments where individuals spend a great amount of their time (i.e. 60% [9]). For example, the use of various services (e.g. banks, restaurants, and post offices) as well as daily (food) shopping, and other activities, such as walking the dog, or jogging may take place in the residential neighborhood.

The majority of studies investigating the relationship between neighborhood characteristics and PA have used self-report methods to measure PA, and included outcomes such as total PA, leisure-time PA, walking, and cycling (see e.g. [10–12]). Although some neighborhood characteristics (e.g. walkability, land use mix) have been consistently associated with increased PA levels [11, 12], it is largely unknown whether neighborhood characteristics may also contribute to objectively measured LPA, and MVPA. Insight in these relationships may provide useful information to develop adequate interventions that aim to increase (a specific) intensity of PA through environmental changes.

In addition, many studies that investigated the role of neighborhood characteristics in PA behavior assessed the role of neighborhood characteristics on total PA levels (see e.g. [13–17]). These PA levels - whether they are walking, overall PA, or leisure time PA – often include both activities within and outside the residential neighborhood. However, this may lead to a conceptual mismatch of PA behavior and environmental exposure [18], which may underestimate the actual physical environment - PA association at the neighborhood level. Thus, there is a need for context-specific PA assessment, where neighborhood characteristics are matched with neighborhood-based PA. One study that examined the association between neighborhood-based PA and neighborhood characteristics, found that higher levels of land use mix, intersection density, and residential population density, and residential housing unit density were positively related to MVPA within a 1 k buffer around the home [19]. Although this provides useful insights, more specificity on the types of objectively measured land use can provide additional and more concrete evidence that contributes to the development of environmental interventions.

Therefore, the aim of this study was to investigate which objective neighborhood characteristics (i.e. types of land uses) are associated with neighborhood-based LPA and MVPA.

## Methods

### Aim, design and setting of the study

This cross-sectional study was part of the PHASE (Physical Activity in public Space Environments) project that aimed to investigate PA behavior in various environments and how environmental settings and their characteristics are related to PA behavior. Participants were randomly recruited from the municipal population register of the cities of Rotterdam and Maastricht, the Netherlands. Recruitment took place in two different cities to compose a study sample with varying environmental exposures (i.e. presence of green, distance to city center, type of buildings, and population density). A more detailed overview of differences in environmental characteristics between Rotterdam and Maastricht, and the four neighborhoods, were provided elsewhere [8]. Adults aged 45–65 years (*N* = 14,889) received an invitation letter to ask them to participate in the study. Adults could register for participation via a website or by telephone. Those who registered (*N* = 516) were contacted by phone or e-mail to plan the distribution of an accelerometer and GPS-device. Trained staff members distributed devices and explained monitor wear to participants (*N* = 406) in community centers close to participants homes. Sheets with a summary of instructions were provided. Data collection took place from April to December 2014. All participants signed informed consent. One participant was excluded from analyses due to insufficient data on the home address. Only participants with sufficient valid accelerometer- and GPS-data were included in analyses. After applying valid data criteria (see below), 308 participants (with a total of 1804 measurement days) could be included in the analyses. The institutional review board of the faculty of Social and Behavioural Sciences of the Utrecht University approved for the study.

### Measures

#### Neighborhood-based PA

Participants were asked to wear an accelerometer and GPS-device, which were attached to an elastic and adjustable belt, during waking hours for 7 consecutive days (except during water-based activities). The Actigraph GT3X+ accelerometer (Actigraph, Pensacola, Florida, FL, USA) was used to measure PA intensity. The epoch length was 5 s. Actilife v6.11.2 (Firmware 2.2.1, Actigraph, Pensacola, Florida, USA) software was used to download accelerometer data. As the study population was middle-aged, and therefore more likely to have longer bouts of sedentary behavior than younger adults non-wear time was defined as episodes of ≥90 min of consecutive zero counts, accepting up to 2 consecutive minutes of 1–100 cpm [20, 21]. Vector magnitude cut-points were used to define light (150–3208 cpm), moderate (3208–8564 cpm) and vigorous (≥8565 cpm) PA [22, 23]. Moderate-vigorous PA was calculated as the sum of moderate and vigorous PA.

BT-Q1000XT GPS-devices (QStarz International Co, Taipei, Taiwan) were used to measure participants’ geographical locations every 5 s. The GPS-device was attached to the same belt the accelerometer was attached to. The QStarz QTravel software (v1.45, Qstarz International Co., Ltd., Taipei, Taiwan) was used to download the data. For each GPS data-point it was determined whether it lay within a 25–400, 25–800, or 25–1600 m Euclidian buffer around participants’ homes. We applied the > 25 m criterion for each neighborhood buffer to exclude the time spent at home. We first calculated the percentage of time spent within each buffer. In addition, GPS- and accelerometer data were date and time linked (using python software), and this combination of data was used to determine the proportion of time spent on light intensity activities (LPA) and moderate-vigorous intensity activities (MVPA) in each buffer. These percentages of time spent on LPA and MVPA in three different buffers (i.e. 400 m, 800 m, and 1600 m) were used as the outcome measures in the analyses.

#### Valid days

A valid day was determined using the 70/80 rule [24]. Therefore, we first determined a measurement day, which is the time during which at least 70% of participants wore the accelerometer devices. For this study, the length of a measurement day was 611 min. The 70/80 rule defines a day to be valid when at least 80% of a measurement day has non-missing counts, which was 488.8 min for this study. Data of participants with at least 4 valid days were included in analyses [25].

#### Objective neighborhood characteristics

The coordinates of the home addresses of participants were uploaded in ArcMap. A 400, 800, and 1600 m Euclidean buffer (drawn around each home address) was used to define participants’ neighborhoods. The proportions of different types of land use (available from Statistics Netherlands, 2012) were calculated for each of these buffers. Nine categories of land use were distinguished: residences, roads, public social and cultural facilities (e.g. educational institutes, churches), shops and food service industry (e.g. shopping centers, cinemas, hotels, restaurants), blue space (i.e. the sum of all proportions of visible surface waters e.g. rivers, lakes, recreational pools in forests, sea), green space (i.e. the sum of all proportions of green such as city parks, allotments, forests, moorland), sports terrain (e.g. football fields, tennis courts, swimming pool, sports hall), and recreational area (e.g. picnic places, zoo). In this paper, neighborhood characteristics thus refer to the proportions of different types of land use (characteristics) in a 400, 800, and 1600 m buffer around participants’ homes (neighborhood). Although these types of land use covered most of the land use types that were found within buffers, there were some types of land use that were not included in the analyses because these proportions were very low. This included for example cemeteries, or dumps.

#### Individual factors

A questionnaire was used to collect data on background variables (e.g. age, gender, ethnicity, and education), the home address, and attitude towards PA. Self-reported highest levels of completed education were classified into three levels: 1) lower education (i.e. no education, primary education, lower professional or intermediate general education); 2) middle education (i.e. intermediate and higher general education); and 3) higher education (i.e. higher professional education and university). Attitude was measured by asking participants to indicate on a 5-point Likert scale to what extent they agreed with four statements: PA is good for me, PA is pleasant, PA is important and PA gives variation. These variables were aggregated into the variable ‘attitude’ by summing the scores of the separate items (Cronbach’s alpha: 0.870, this value did not increase if items were deleted).

### Statistical analyses

All analyses were performed using SPSS 23.0 for windows (IBM SPSS Inc., Armonk, NY). Descriptive statistics were used to present data on population- and neighborhood characteristics, and the amounts of time spent within neighborhood buffers. To assess the role of neighborhood characteristics (independent variables) in neighborhood-based LPA and MVPA (outcome variables), bootstrapped multilevel linear regression analyses were performed. Regressions were bootstrapped because the outcome variables were not normally distributed and neither log transformations nor taking the square root led to normal distributions. Multilevel analyses were used to consider the multilevel structure of the data: days were organized within respondents (and days of one respondent are more similar to each other than to those of other respondents). Analyses were adjusted for age, gender, BMI (Body Mass Index), education, ethnicity, having a car, having children, dog ownership, city of residence (Rotterdam or Maastricht), and attitude towards PA.

## Results

### Descriptive statistics

Of the total study population (*N* = 308), a little more than half was female (Table 1). Adults were on average 56.4 (SD 6.2) years, over 60 % were employed, most adults had a middle or higher education, and more than 80% of the population was native Dutch. About 1/3 of the study population had at least one child, and approximately 1/5 had a dog. The most common type of land use of the buffers surrounding participants’ homes was residences (Table 2).

**Table 1:** Descriptive statistics of the study population (*N* = 308)

*Individual factors*	
Age in years *Mean (± SD)*	56.4 (± 6.2)
Female *%*	54.9
BMI *%*	
Healthy weight	52.9
Overweight	37.0
Obesity	10.1
Education *%*	
Low	4.2
Middle	53.2
High	40.9
Missing	1.6
Ethnicity *%*	
Autochthonous	84.4
Western immigrants	6.8
Non-western immigrants	7.5
Missing	1.3
Having a dog *%*	
Yes	18.8
Missing	0.6
Having children *%*	
Yes	33.1
Having a car *%*	
Yes	82.8
Missing	0.6
City *%*	
Rotterdam	38.0
Maastricht	62.0
Attitude *(score)*	
Mean (± SD)	18.0 (2.4)
Missing (%)	0.3

Note: *SD* standard deviation, *BMI* Body Mass Index, *IQR* interquartile range

^a^This includes the time spent at home. ^b^Excluding the time spent at home

**Table 2:** Proportions of participants’ (N = 308) with certain neighborhood characteristics (% land use) in different buffers surrounding their homes

	400 m buffer				800 m buffer				1600 m buffer			
	Mean	(± SD)	Median	(IQR)	Mean	(± SD)	Median	(IQR)	Mean	(± SD)	Median	(IQR)
Residences	64.8	(14.1)	67.8	(21.2)	52.5	(13.0)	54.8	(19.0)	38.2	(9.2)	37.9	(17.1)
Roads	5.1	(3.5)	4.4	(3.4)	5.5	(2.3)	5.6	(3.5)	5.5	(2.0)	5.7	(1.9)
Shops and foodservice industry	4.8	(7.0)	2.7	(6.2)	4.3	(6.5)	2.3	(3.1)	3.8	(6.5)	2.0	(3.5)
Public social-cultural facilities	4.0	(6.1)	1.9	(5.8)	4.2	(4.4)	2.8	(5.3)	4.6	(2.9)	4.1	(4.6)
Green space	9.8	(10.1)	7.2	(15.0)	13.8	(12.6)	10.3	(14.8)	20.1	(12.9)	17.5	(17.8)
Blue space	2.3	(4.8)	0.0	(2.4)	5.1	(7.8)	0.0	(8.2)	7.3	(8.8)	3.0	(14.2)
Sports terrain	2.4	(5.7)	0.0	(2.5)	3.6	(5.0)	2.3	(2.4)	4.3	(3.1)	3.0	(3.6)
Recreational area	0.9	(3.3)	0.0	(0.0)	1.2	(2.7)	0.0	(0.9)	1.1	(1.4)	0.4	(1.5)

Note: *SD* standard deviation, *IQR* interquartile range. Most land use variables were not normally distributed, hence the median is presented. As for some variables the median was 0, the mean was also presented for interpretation

Participants spent on average 29.1% of a measurement-day in LPA, and 5.7% in MVPA (Table 3). Participants spent more time outside the 400, 800, and 1600 m buffers than within the buffers. The average percentage of LPA and MVPA within the buffers is approximately similar to LPA and MVPA outside the buffers.

**Table 3:** Daily percentages of total time, light PA, and moderate-vigorous PA spent in different buffers

	Total time %		LPA %		MVPA %	
	*Median*	*IQR*	*Median*	*IQR*	*Median*	*IQR*
Total			29.1	(22.6; 35.9)	5.7	(3.4; 8.9)
At home (0-25 m)	40.5	(19.1; 62.8)	27.6	(19.8; 36.0)	3.9	(2.4; 6.6)
Within a 25-400 m buffer	11.6	(4.9; 24.0)	27.9	(19.3; 38.2)	4.5	(2.1; 9.6)
Within a 25-800 m buffer	13.8	(6.4; 28.6)	28.6	(20.1; 38.9)	4.6	(2.2; 10.0)
Within a 25-1600 m buffer	19.8	(9.4; 36.3)	30.0	(21.4; 38.6)	5.1	(2.5; 10.3)
> 1600 m from the home	24.2	(5.1; 55.7)	28.6	(21.9; 35.9)	5.0	(3.0; 8.2)

Note: Percentages represent daily averages of measurement days. Percentages in columns (e.g. at home, within 25-400 m, and > 400 m) do not necessarily add up to 100% because medians are presented. Medians were presented due to non-normality. *LPA* Light PA, *MVPA* Moderate-Vigorous PA

### The role of neighborhood characteristics in LPA and MVPA within the neighborhood

Various objective neighborhood characteristics were significantly associated with LPA and MVPA within the neighborhood (Table 4). Neighborhood characteristics that were associated with LPA were different from the neighborhood characteristics that were associated with MVPA. Also, different significant associations were found for buffers of different sizes.

**Table 4:** Associations between neighborhood characteristics and percentage of LPA and MVPA within the neighborhood

	*B*	*95% CI*	*p*	*B*	*95% CI*	*p*
	% LPA within a 25-400 m buffer			% MVPA within a 25-400 m buffer		
Neighborhood characteristics within a 25-400 m buffer						
Intercept	26.18			9.54		
Residences	0.01	(− 0.07; 0.09)	0.847	**0.05**	**(0.01; 0.10)**	**0.010**
Roads	**0.20**	**(−0.01; 0.42)**	**0.032**	0.02	(− 0.13; 0.16)	0.802
Shops and foodservice industry	0.04	(−0.09; 0.16)	0.481	**0.15**	**(0.07; 0.23)**	**0.001**
Sports terrain	0.04	(−0.09; 0.17)	0.468	**0.08**	**(0.00; 0.17)**	**0.023**
Public social-cultural facilities	0.03	(−0.10; 0.18)	0.590	**0.06**	**(−0.01; 0.12)**	**0.042**
Recreational area	−0.17	(− 0.39; 0.06)	0.080	**− 0.12**	**(− 0.23; − 0.03)**	**0.005**
Green space	− 0.08	(− 0.19; 0.02)	0.083	**0.11**	**(0.02; 0.19)**	**0.001**
Blue space	0.10	(−0.08; 0.27)	0.187	**0.19**	**(0.10; 0.28)**	**0.001**
	% LPA within a 25-800 m buffer			% MVPA within a 25-800 m buffer		
Neighborhood characteristics within a 25-800 m buffer						
Intercept	25.96			7.88		
Residences	0.05	(−0.03; 0.12)	0.172	0.03	(−0.02; 0.09)	0.166
Roads	−0.05	(−0.36; 0.22)	0.682	−0.00	(− 0.19; 0.19)	0.983
Shops and foodservice industry	0.11	(−0.05; 0.25)	0.118	**0.09**	**(−0.00; 0.18)**	**0.034**
Sports terrain	−0.02	(−0.17;0.13)	0.707	**0.15**	**(0.04; 0.27)**	**0.004**
Public social-cultural facilities	−0.01	(−0.19; 0.18)	0.911	**0.12**	**(0.04; 0.21)**	**0.003**
Recreational area	**−0.30**	**(−0.63; 0.04)**	**0.047**	−0.16	(− 0.35; 0.07)	0.095
Green space	**−0.09**	**(−0.18; 0.00)**	**0.032**	0.04	(−0.02; 0.11)	0.136
Blue space	0.12	(−0.02; 0.27)	0.057	**0.08**	**(−0.01; 0.16)**	**0.046**
	% LPA within a 25-1600 m buffer			% MVPA within a 25-1600 m buffer		
Neighborhood characteristics within a 25-1600 m buffer						
Intercept	29.35			11.53		
Residences	0.03	(−0.08; 0.16)	0.567	−0.00	(−0.09; 0.08)	0.905
Roads	−0.31	(−1.29; 0.69)	0.465	−0.40	(−1.10; 0.28)	0.166
Shops and foodservice industry	−0.14	(−0.43; 0.15)	0.289	−0.02	(− 0.19; 0.16)	0.824
Sports terrain	−0.10	(−0.52; 0.34)	0.591	0.04	(−0.27; 0.35)	0.787
Public social-cultural facilities	0.16	(−0.32; 0.68)	0.487	**0.38**	**(0.03; 0.72)**	**0.015**
Recreational area	0.23	(−0.61; 0.99)	0.508	0.07	(−0.42; 0.65)	0.787
Green space	**−0.08**	**(−0.16; 0.01)**	**0.045**	0.02	(−0.04; 0.08)	0.394
Blue space	−0.06	(−0.27; 0.15)	0.497	−0.06	(− 0.19; 0.07)	0.315

Note. All models were adjusted for: age, gender, BMI, education, ethnicity, having a car, having children, dog ownership, city, and attitude towards PA. *LPA* Light PA, *MVPA* Moderate-Vigorous PA, *CI* Confidence Interval

Bolded text highlights significant associations

The proportion of roads was positively associated with LPA within a 400 m buffer. The proportions of recreational area and green space were negatively associated with LPA within an 800 m buffer, and the proportion of green space was negatively associated with LPA within a 1600 m buffer.

With regard to MVPA, positive associations were found between the proportions of residences, shops and foodservice industry, sports terrain, public social-cultural facilities, green space, and blue space and MVPA within a 400 m buffer, whereas the proportion of recreational area was negatively associated with MVPA within a 400 m buffer. The proportions of shops and foodservice industry, sports terrain, public social-cultural facilities, and blue space were positively associated with MVPA within an 800 m buffer. Further, the proportion of public social-cultural facilities was positively associated with MVPA within a 1600 m buffer.

## Discussion

This study found significant associations between objective neighborhood characteristics and neighborhood-based LPA and MVPA, also when adjusted for an extensive set of individual factors, including attitude towards PA. This makes it less likely that the correlation between neighborhood characteristics and neighborhood-based PA is purely a matter of selection (i.e. that those with a more favorable attitude towards PA, and practicing more PA, chose to reside in neighborhoods that facilitate PA), which may indicate that a causal mechanism underlies the correlations found in this study.

The current study showed that more neighborhood characteristics were associated with MVPA than with LPA. A possible explanation for this finding may be that LPA is often part of everyday activities (e.g. household activities, walking), which are integrated in adults’ daily activity patterns. That is, these activities may be more likely to occur in any case, whereas MVPA may require more planning, skills, motivation, and specific facilities or environmental features.

Additionally, most effects are found for the smallest buffer around the home (i.e. 400 m buffer), whereas less (i.e. for MVPA) or even negative (i.e. for LPA) associations were found for the larger buffers. This suggests that objective neighborhood characteristics may be of particular importance for PA in the area directly surrounding adults’ homes. Further, these findings emphasize that size of a buffer matters when assessing the physical environment – PA relationship. Hence, future studies should consider the use of multiple buffers when assessing the relationship between neighborhood characteristics and PA, or choose a buffer that fits specific policy or urban design aims.

The importance of environmental characteristics close to individuals’ homes seems to apply in particular to natural environments (i.e. green and blue spaces), as the positive associations found for MVPA within a 400 m buffer disappear when buffer size increases. Moreover, negative associations were found between green space and LPA within the 800 and 1600 m buffers. A possible explanation for this may be that adults are more familiar with green and blue spaces within a shorter distance from their home than with green and blue spaces further away. Also, the use of green and blue spaces on short distance to the home is easier to integrate with obligatory activities during the day. With the current findings, this study expands existing literature that found that amount and size of, and distance to urban green space, play an important role in stimulating PA behavior [26, 27], by demonstrating that this effect emerges via PA close to the residence. Also, the positive effects of blue spaces on physical activities such as walking (the dog), jogging or cycling at the water’s edge [28], apparently occur mostly on short distance to the home.

Proportions of roads was the only type of land use that was positively associated with LPA, and only within a 400 m buffer. An explanation for this may be that an increased proportion of roads is related to increased walkability and connectivity, and a reduced distance to side-walks or trails; factors that have been positively related to walking (i.e. an important source of LPA [7]) and PA in previous studies (see e.g. [10–12]). Also, findings that higher proportions of sports terrain, public social-cultural facilities, and shops and foodservice industry were positively associated with MVPA are in line with existing literature that showed, although not at the level of neighborhood-based MVPA, that sports facilities [29] and presence and accessibility of shops and other facilities (see e.g. [10, 30]) were associated with (MV)PA.

Perhaps less expected -at first sight- was the finding that a higher proportion of recreational areas within one’s residential neighborhood was associated with lower levels of LPA within an 800 m buffer and MVPA within a 400 m buffer. However, in this study, the land use category of ‘recreational area’ did not include parks or sports facilities, which belonged to other types of land uses (i.e. green space and sports facilities, respectively). In the current study, ‘recreational area’ refers to places such as zoos, amusement parks, open-air museums, playgrounds, and picnic places. Such places likely facilitate mostly sedentary behavior (e.g. social activities) and not so much LPA or MVPA, especially among this middle-aged study sample.

### Strengths and limitations

The use of accelerometers and GPS-devices provided accurate and detailed information on neighborhood-based LPA and MVPA levels of adults. In addition, the use of objective land use information added to existing literature that mostly reported perceived environmental factors and/or observed environmental factors [12]. Furthermore, we included an extensive set of individual factors in analyses to correct for possible confounding effects (e.g. those with certain individual characteristics may select PA promoting neighborhoods).

The use of accelerometers and GPS-devices also has limitations. For example, upper-body movements are less well recorded by the accelerometer and water-based activities (i.e. swimming) could not be measured [31]. GPS-devices may suffer from canyoning (i.e. high buildings, or trees interfere with satellite communication), but the QStarz GPS-device that was used in this study has shown to have a high accuracy even in urban canyons [32]. Moreover, the use of GPS-devices and accelerometers often comes with relatively smaller study populations as compared to the use of questionnaires. However, although the response rate of this study was relatively low, our final study sample was comparable to other studies [12].

Finally, it is likely that a selective sample, i.e. adults who are interested in PA and like being active, responded to the invitation to participate in this study. Hence, more active adults may be included in this study, which may have led to an overestimation of PA levels. In addition, when comparing the figures of our sample with those of the total Dutch population, we see that overweight and obese adults, lower educated adults, and (non-) western immigrants were underrepresented in the study sample. Since the literature shows inconsistent findings on the correlations of these characteristics with PA (i.e. positive, negative, as well as null associations – see e.g. [33, 34]), it remains unclear whether these under representations affected our results.

The inclusion of two different cities in the Netherlands contributed to differences in exposure to neighborhood environmental characteristics. Therefore, the findings of this study are more likely to be representative for the Netherlands. However, the findings of this study may not be applicable to other counties as urban design and environmental characteristics of residential areas (e.g. walking and cycling facilities, size of green spaces) may be very different between countries. In addition, the results of this study were found for a specific age group and future research is needed to assess what objectively measured neighborhood characteristics are related to neighborhood-based LPA and MVPA of other age groups (e.g. youth or older adults).

Due to the cross-sectional design of this study, only associations could be assessed and not causal relations. Future research should apply longitudinal, and preferably pre-posttest designs to investigate causal relationship between such neighborhood characteristics and PA.

## Conclusions

This study responded to the need for more context-specific PA assessment by providing new insights in the role of objective neighborhood characteristics in neighborhood-based LPA and MVPA. Two main conclusions can be drawn from this study: 1) objective neighborhood characteristics play an important role in neighborhood-based PA, also when adjusted for socio-demographic factors and attitude towards PA. Hence, associations between the residential environment and PA found in previous studies can at least partly be explained by the effects on PA in the neighborhood, and 2) these neighborhood characteristics seem to be of particular importance for PA in the areas close to adults’ homes (i.e. the smallest buffer around the home). Hence, size of the buffer matters when assessing the relationship between the residential environment and PA. Longitudinal pre-posttest study designs are necessary to assess the causality of the associations between objective neighborhood characteristics and objectively measured PA.

## Abbreviations

BMI: Body Mass Index
CI: Confidence Interval
IQR: Interquartile Range
LPA: Light Physical Activity
MVPA: Moderate to Vigorous Physical Activity
PA: Physical Activity
SD: Standard Deviation
